# Neuroimaging young children and associations with neurocognitive development in a South African birth cohort study

**DOI:** 10.1016/j.neuroimage.2020.116846

**Published:** 2020-10-01

**Authors:** Catherine J. Wedderburn, Sivenesi Subramoney, Shunmay Yeung, Jean-Paul Fouche, Shantanu H. Joshi, Katherine L. Narr, Andrea M. Rehman, Annerine Roos, Jonathan Ipser, Frances C. Robertson, Nynke A. Groenewold, Diana M. Gibb, Heather J. Zar, Dan J. Stein, Kirsten A. Donald

**Affiliations:** a Department of Paediatrics and Child Health, Red Cross War Memorial Children’s Hospital, University of Cape Town, South Africa; b Department of Clinical Research, London School of Hygiene & Tropical Medicine, UK; c Neuroscience Institute, University of Cape Town, South Africa; d Department of Psychiatry, University of Cape Town, South Africa; e Departments of Neurology, Psychiatry and Biobehavioral Sciences, University of California Los Angeles, CA, USA; f MRC Tropical Epidemiology Group, London School of Hygiene & Tropical Medicine, London, UK; g SU/UCT MRC Unit on Risk and Resilience in Mental Disorders, Department of Psychiatry, Stellenbosch University, South Africa; h Division of Biomedical Engineering, Department of Human Biology, University of Cape Town, South Africa; i Cape Universities Brain Imaging Centre (CUBIC), Cape Town, South Africa; j MRC Clinical Trials Unit, University College, London, UK; k SAMRC Unit on Child & Adolescent Health, University of Cape Town, South Africa; l SU/UCT MRC Unit on Risk and Resilience in Mental Disorders, University of Cape Town, South Africa

**Keywords:** Neuroimaging, Children, Africa, Cortical surface area, Cortical thickness, Cognition

## Abstract

Magnetic resonance imaging (MRI) is an indispensable tool for investigating brain development in young children and the neurobiological mechanisms underlying developmental risk and resilience. Sub-Saharan Africa has the highest proportion of children at risk of developmental delay worldwide, yet in this region there is very limited neuroimaging research focusing on the neurobiology of such impairment. Furthermore, paediatric MRI imaging is challenging in any setting due to motion sensitivity. Although sedation and anesthesia are routinely used in clinical practice to minimise movement in young children, this may not be ethical in the context of research. Our study aimed to investigate the feasibility of paediatric multimodal MRI at age 2–3 years without sedation, and to explore the relationship between cortical structure and neurocognitive development at this understudied age in a sub-Saharan African setting. A total of 239 children from the Drakenstein Child Health Study, a large observational South African birth cohort, were recruited for neuroimaging at 2–3 years of age. Scans were conducted during natural sleep utilising locally developed techniques. T1-MEMPRAGE and T2-weighted structural imaging, resting state functional MRI, diffusion tensor imaging and magnetic resonance spectroscopy sequences were included. Child neurodevelopment was assessed using the Bayley-III Scales of Infant and Toddler Development. Following 23 pilot scans, 216 children underwent scanning and T1-weighted images were obtained from 167/216 (77%) of children (median age 34.8 months). Furthermore, we found cortical surface area and thickness within frontal regions were associated with cognitive development, and in temporal and frontal regions with language development (beta coefficient ≥0.20). Overall, we demonstrate the feasibility of carrying out a neuroimaging study of young children during natural sleep in sub-Saharan Africa. Our findings indicate that dynamic morphological changes in heteromodal association regions are associated with cognitive and language development at this young age. These proof-of-concept analyses suggest similar links between the brain and cognition as prior literature from high income countries, enhancing understanding of the interplay between cortical structure and function during brain maturation.

## Highlights

• MRI data are challenging to acquire in the early years of life.
• Paediatric MRI without sedation is feasible in sub-Saharan Africa, with 77% success.
• The Drakenstein Child Health study has novel MRI data of South African children.
• Morphological features of the cortex associate with neurocognitive development.
• Structure-cognition relationships in heteromodal association regions at 2–3 years.

## 1 Introduction

The first three years of life represent the most extensive period of brain growth and synapse development, where critical neural pathway development and network maturation occur (Hermoye et al., 2006; Ouyang et al., 2019; Knickmeyer et al., 2008). This early brain development shapes each child’s future potential and is critical to later educational outcomes and human capital (Walker et al., 2011; Daelmans et al., 2017). The United Nations Sustainable Development Goals (SDGs, https://sustainabledevelopment.un.org) have focused the world on the importance of children thriving; however, 43% of children under 5 years are at risk of failing to reach their developmental potential worldwide. The majority of these children live in low and middle-income countries (LMIC); sub-Saharan Africa (SSA) has the highest proportion of children at risk of developmental delay (Black et al., 2017).

Magnetic resonance imaging (MRI) has revolutionised our ability to examine brain structure, function and connectivity, and emergent technologies have allowed increasingly sophisticated investigation into neuroanatomy and neurocircuitry. MRI has many advantages over other imaging modalities including high image resolution without radiation exposure, offering a safe tool for investigating early brain development in young children and the neurobiological mechanisms behind developmental delay. However, MRI studies of young children in LMICs are lacking (Azhari et al., 2019) and very little research has been performed to assess the link between early brain structure and cognitive development in SSA (Paterson et al., 2006).

Studies from high income countries have used MRI to examine brain development in neonates, school-aged children, and adolescents (Wierenga et al., 2018; Hagler et al., 2019). However, only a few have investigated brain structure and function in preschool children between the ages of 1–3 years when cortical maturation is most rapid, despite recognition of the importance of this period in terms of structural and functional development (Gilmore et al., 2018). Beyond early infancy, paediatric imaging is difficult due to the MRI requirement of lying still in an enclosed space for a prolonged period of time (Barkovich et al., 2019) leading to technical and practical challenges (Almli et al., 2007; Raschle et al., 2012; Thieba et al., 2018). This is particularly notable in preschool children who are not able to understand the reasons for this requirement (Raschle et al., 2012; Thieba et al., 2018) and are not responsive to mock scanner training which can be effective for older children (Thieba et al., 2018). Although sedation and anesthesia are frequently used in clinical practice, there are risks associated with these approaches that have ethical implications for their use in research (Edwards and Arthurs, 2011; Jevtovic-Todorovic et al., 2013; Cote et al., 2000; Sammons et al., 2011). These techniques may also dynamically affect brain signal during functional imaging (Jevtovic-Todorovic et al., 2013).

Previous studies from high income countries in infancy and later childhood have found that distinct brain areas are associated with various neurodevelopmental functions, including areas of the frontal lobe associated with cognitive development (Paterson et al., 2006; Lyall et al., 2015; Shaw et al., 2006) and frontal and temporal regions associated with language (Imada et al., 2006; Dehaene-Lambertz et al., 2010). There is a need for studies to investigate the brain structure-cognition relationship across different socio-cultural contexts (Azhari et al., 2019) and in LMICs where the majority of children at-risk of developmental impairment reside (Tomlinson et al., 2014). Neuroimaging from a young age may provide insight into early neurodevelopment processes as well as the relationships with current and future neuropathology (Gilmore et al., 2018; Jahanshad et al., 2015; Gao et al., 2019).

The Drakenstein Child Health Study is a large population-based birth cohort study in the Western Cape of South Africa investigating the early life determinants of child health (Stein et al., 2015; Zar et al., 2015). This cohort study offered a unique opportunity to investigate associations between early brain structure and neurodevelopment. In this prospective study, we aimed firstly to establish the feasibility of performing paediatric multimodal MR scanning during natural sleep at age 2–3 years in a sub-Saharan African setting; secondly to assess the association between scan success and neurodevelopment; and thirdly to explore the relationships between regional cortical structure and cognitive and language development at this young age, important predictors of later cognitive outcome**.**

## 2 Materials and methods

### 2.1 Study design

The Drakenstein Child Health Study (DCHS) is an observational population-based birth cohort in South Africa (Stein et al., 2015; Zar et al., 2015). Women were recruited during pregnancy and mother-child pairs are followed up longitudinally. Neuroimaging was performed as a prospective nested study.

### 2.2 Study setting

The DCHS study is located in the Drakenstein sub-district, a peri-urban area 60 ​km outside Cape Town, Western Cape, South Africa. This sub-district has a high burden of infectious diseases and psychosocial stressors representative of many other LMICs (Stein et al., 2015). Pregnant women were recruited into the DCHS from two public sector primary health care clinics: Mbekweni (serving a predominantly black African community) and TC Newman (serving a mixed ancestry community) between March 2012 and March 2015.

### 2.3 Participants

#### 2.3.1 Drakenstein Child Health Study

Mothers were enrolled into the DCHS at 20–28 weeks’ gestation while attending routine antenatal care using an unfiltered approach to ensure the cohort was representative of the local population. Pregnant women were eligible for the study if they were 18 years or older, attended one of the recruitment clinics and intended to remain in the area. Mothers provided written informed consent at enrolment and are re-consented annually. Consent was done in the mother’s preferred language: English, Afrikaans or isiXhosa. Between May 2012 and September 2015, there were 1143 live infants in the umbrella DCHS. In the catchment area of the Drakenstein sub-district where the majority of births occur at Paarl Hospital, the study enrolled approximately 10% of births (Donald et al., 2018). This represents approximately 0.03% of all births in South Africa during this time period (Stats SA, 2016).

#### 2.3.2 Neuroimaging sub-study

A sub-group of mother-child pairs were selected from the DCHS cohort for neuroimaging at 2–3 years of age with a pilot phase from July to December 2015, and the main study from January 2016 to September 2018. A total of 239 mother-child pairs were invited to attend for neuroimaging when the child turned 2 years who were known to be currently active in the cohort, staying in the study area, and had none of the following exclusion criteria: (i) Medical comorbidity (genetic syndrome, neurological disorder, or congenital abnormality); (ii) Gestation <36 weeks; (iii) Low Apgar score (<7 ​at 5 ​min); (iv) Neonatal intensive care admission; (v) Maternal use of illicit drugs during pregnancy; (vi) Child HIV infection. Children who underwent MRI in the neonatal period were prioritised; methods are described in full elsewhere (Donald et al., 2018). Children were selected for neuroimaging based on risk factor exposure to ensure adequate representation, and a randomly selected comparison group frequency matched by age and sex. Written informed consent was obtained from the parent.

### 2.4 Demographics

Sociodemographic data were collected in interviews at 28–32 weeks’ gestation by trained study staff using validated questionnaires (Myer et al., 2008). Measures of socioeconomic status (SES) included household income, maternal education and employment. In order to adequately capture variability of SES within this setting, we also standardised these measures along with an asset index and created an aggregate measure of SES (Myer et al., 2008), divided into quartiles. Study staff attended births and birth data were prospectively collected or abstracted from hospital records. Child gestational age was calculated from antenatal ultrasound where available, or symphysis-fundal height or maternal report of last menstrual period.

### 2.5 Clinical developmental outcomes

Child development was assessed using the Bayley Scales of Infant and Toddler Development, Third Edition (BSID-III) at 2 years of age (Bayley, 2006; Ballot et al., 2012). The BSID-III assessment is one of the most comprehensive tools to assess early child development. It is sensitive to subtle developmental delay across cognitive, language and motor scales (Bayley, 2006; Albers and Grieve, 2007), and has been validated in South Africa (Ballot et al., 2012; Rademeyer and Jacklin, 2013). The BSID-III was administered by trained assessors who used direct observation to assess child development and gave language prompts in the child’s preferred language (Afrikaans or isiXhosa). Assessments were standardised to ensure concordance. Scoring was done according to the manual and BSID specialized software which produces norm-referenced scores across subscales. Standardised composite scores were calculated (mean 100, standard deviation [SD] 15) using normative values from a US reference population as per the assessment guidance to allow comparison across domains. Cognitive and language composite scores were included for this analysis as measures of neurocognitive function.

### 2.6 Neuroimaging

#### 2.6.1 Data acquisition

Neuroimaging was performed at the Cape Universities Brain Imaging Centre (CUBIC). The pilot phase took place at Tygerberg Hospital from July to December 2015 on a 3T ​Siemens Allegra MRI scanner (Erlangen, Germany), where our group had experience with scanning neonates (Donald et al., 2015). From January 2016 to September 2018 the main neuroimaging sub-group (excluding the pilot group) had scanning performed at Groote Schuur Hospital on a research-dedicated 3T Siemens Skyra 70 ​cm diameter bore whole body MRI scanner (Siemens, Erlangen, Germany) (http://www.cubic.uct.ac.za). A 32-channel head coil optimised for young children was used with stabilising cushions to reduce head movement during scans. The full scan protocol included 1) MEMPRAGE T1 and T2-weighted structural imaging; 2) resting state functional imaging; 3) single voxel magnetic resonance spectroscopy; and 4) diffusion tensor imaging. The total scan duration was approximately 1 ​h (58 ​min 38 ​s) with slight variation depending on shimming. See Table 1 for sequence parameters and measures. Processing and analysis procedures described below are focused on the structural scans only.

**Table 1:** Imaging modalities and acquired parameters.

Sequence	Measures	Parameters: Siemens Skyra sequences
3D T1-weighted MEMPRAGE (Multi-Echo Magnetization Prepared Rapid Acquisition Gradient Echo)	Subcortical and cortical tissue volumes; Surface-wise measures including cortical thickness, surface area and gyrification.	Sagittal orientation; Repetition time (TR) ​= ​2530 ​ms; echo time (TE) ​= ​1.69, 3.54, 5.39, 7.24 ​ms; flip angle ​= ​7.0°; voxel size 1.0 ​× ​1.0 ​× ​1.0 ​mm^3^; inversion time (TI) ​= ​1100 ​ms; field of view (FOV) ​= ​224 ​× ​224 ​× ​176 mm; 176 slices, 1.0 ​mm thick. Scan time: 5min21s.
Resting state blood-oxygen-level dependent (BOLD) echo planar imaging (EPI)	Resting brain networks.	TR 2000 ​ms; TE 30 ​ms; flip angle ​= ​77°, 33 slices, slice thickness 4 ​mm; slice gap 1 ​mm, voxel size 3.4 ​× ​3.4 ​× ​4.0 ​mm. FOV ​= ​220 ​× ​220mm, Scan time 8min04s.

Single voxel PRESS (Point RESolved Spectroscopy) magnetic resonance spectroscopy (MRS)	Relative metabolite concentrations of phosphocreatine (Cr ​+ ​PCr), glutamate with glutamine (Glx), glutamate (Glu), n-acetyl-aspartate with n-acetyl-aspartyl-glutamate (NAA ​+ ​NAAG), n-acetyl-aspartate (NAA), choline containing metabolites (glycerophosphocholine ​+ ​phosphocholine [GPc ​+ ​PCh]), and ​myo-inositol (mI).	*Voxel 1:* Midline parietal grey matter. *Voxels 2 and 3*: Left and right parietal white matter. TR ​= ​2000 ​ms, TE ​= ​30 ​ms (128 averages) with water references (TE ​= ​30, 75, 100, 144, 500, 1000 ​ms); Voxel size 25 ​× ​25 ​× ​25 mm^3^. Scan time: ~6 ​min total per voxel with water references.
Diffusion Tensor Imaging (DTI)	Fractional anisotropy (FA), mean diffusivity (MD), radial diffusivity (RD) and axial diffusivity (AD).	A pair of diffusion-weight datasets with opposite phase encoding (anterior-posterior and posterior-anterior) were acquired using 30 noncollinear gradient directions with DWfactor *b* ​= ​1000 ​s/mm^2^, and one non-DW *b* ​= ​0s mm^2^ (*b*_*0*_) acquisition); TR ​= ​7800 ​ms; TE ​= ​92 ​ms; voxel size ​= ​1.8 ​× ​1.8 ​× ​2.0 ​mm^3^, FOV ​= ​230 x 230 x 121 ​mm, slice thickness 2.0 ​mm, Scan time: 2 ​× ​8min36s.

3D Sagittal T2-weighted structural imaging	Subcortical and cortical tissue volumes.	TR ​= ​3200 ​ms; TE ​= ​409 ​ms; FOV ​= ​230 ​× ​230 mm; voxel size ​= ​0.9 ​× ​0.9 ​× ​1.0 ​mm^3^; 160 slices, 1.0 ​mm thick. Scan time: 3min7s

The scans were undertaken during natural sleep utilising locally developed techniques: imaging was conducted after lunch or in the evening to coincide with routine sleep times. Mothers were asked to keep the child awake prior to the scan. On arrival, a full explanation of the MRI process was given, informed consent was taken from the parent/guardian and an MRI safety screening questionnaire was administered. Children were directed to a separate playroom equipped with toys and picture books with a member of the study team to maximise comfort and allow acclimatisation to an unfamiliar environment. The team adopted a child-friendly approach throughout the whole process to put the children at ease and incorporated play to reduce anxiety (Raschle et al., 2012). Children were encouraged to play quietly to encourage sleeping afterwards, and anthropometric data collection was integrated into this routine. The child and caregiver were given a warm meal, and the child was given melatonin (dosage 3–6 ​mg) (Ibekwe et al., 2017; Paediatric Formulary Committee, 2016) with yoghurt to improve the taste to promote sleep-initiation (Johnson et al., 2002). The children were wrapped warmly and encouraged to sleep in their natural position – either swaddled on their mother’s back (common practice in this community) or lying on a bed in a low-lit room. Once the child had fallen into deep sleep, he/she was carried into the scanner, positioned carefully and ear protection was fitted. The scanner was padded with pillows and blankets and a trained study staff member remained in the scanner room throughout the scan to alert the radiographer if the child woke.

During acquisition, images were checked in real-time and if a substantial artefact was observed (most commonly due to movement), and there was sufficient time, then that specific sequence was repeated. Similarly, if the child woke during the scan protocol then he/she was encouraged to sleep again so the protocol could be completed. If further sleep was not possible, with parental consent the child was brought back on a different day to attempt the scan again, up to a maximum of three times.

#### 2.6.2 Scan reporting

Structural sequences were reviewed by a radiologist to check for clinical incidental findings and a formal report was provided to the study team. Any incidental findings were discussed with a paediatric neurologist. Relevant findings were discussed with the child’s parents, and referred for management through established local clinical pathways as appropriate.

#### 2.6.3 Processing and quality control overview

Imaging data processing included 1) quality checking to prepare the data and correct for motion artefact; 2) processing as per each individual modality to extract the relevant outcome measures; and 3) statistical analysis. Below we outline the processing conducted on the T1-weighted scans.

##### 2.6.3.1 T1-weighted MR images processing and quality control

After the image acquisition, all T1-weighted MR images were processed using FreeSurfer version 6.0 software (http://surfer.nmr.mgh.harvard.edu/) utilising the automated techniques for cortical reconstruction and volumetric segmentation (Fischl et al., 2002, 2004; Dale et al., 1999; Desikan et al., 2006). Structural T1 images were first converted from DICOM to NIfTI format. Scans were then processed through the FreeSurfer programme using the recon-all command at the local supercomputing cluster at the Centre for High Performance Computing (CHPC, Cape Town) (https://www.chpc.ac.za). The pipeline involved skull stripping, B1 bias field correction, normalisation, grey-white matter segmentation, surface atlas registration and extraction, and automated cortical reconstruction producing regional and total brain volumes, and anatomical measures including cortical surface area and cortical thickness (Fischl and Dale, 2000). Cortical regions-of-interest (ROIs) (surface area and thickness) were extracted for analysis.

The T1-MEMPRAGE images were checked for movement and completeness. Images were visually quality checked for movement artefact. FreeSurfer outputs were also visually inspected for errors in segmentation of cortical and subcortical structures. Overall, FreeSurfer processing was not possible in one case, and one child had an artefact that failed correction after processing. There were no errors of segmentation of cortical structures, however, two children were found to have incidental findings on clinical report Using the ENIGMA pipeline (http://enigma.ini.usc.edu/protocols/imaging-protocols/), subjects were reviewed if ROIs in the final output were classified as extreme outliers (Nwosu et al., 2018; ENIGMA, 2019).

### 2.7 Ethics

The DCHS was approved by the Faculty of Health Sciences, Human Research Ethics Committee, University of Cape Town (401/2009) and by the Western Cape Provincial Health Research Committee. The imaging was approved by the Faculty of Health Sciences, Human Research Ethics Committee, University of Cape Town (525/2012) and the London School of Hygiene & Tropical Medicine (11903).

### 2.8 Statistical analyses

#### 2.8.1 Scan success

Children were categorised as having a full scan (completed acquisition of all sequences), part successful scan (1–4 sequences), or no scan to quantify scan success. Developmental outcomes, as measured by the BSID-III, were calculated as standardised composite scores (continuous measures) and categorised into delay variables (using a standard cut-off of ​< ​-1 SD from the BSID-III reference mean) reported as means and standard deviations and proportions respectively. Child clinical neurodevelopment, age and sex were associated with scan success using ANOVA.

#### 2.8.2 Cortical structure and neurocognitive development

Analyses were conducted on n ​= ​146 children who had complete data available for all relevant independent variables (age, sex, ROI) and at least one dependent clinical development measure (cognitive or language development). Sociodemographic and developmental outcomes were expressed as mean (SD) for continuous data and frequencies (%) for categorical data. Comparisons were made with the full DCHS cohort to assess generalisability using descriptive statistics (χ^2^ test).

Cortical surface area and cortical thickness measurements were standardised and associated with composite developmental outcomes (cognitive and language development) from the BSID-III at 2 years. Multiple linear regression was used to model regional associations with neurodevelopmental outcomes, where cognitive development was the dependent variable, and cortical surface area or thickness were independent variables. Given the substantial variability of child developmental trajectories; the rapid development during this time meaning cognitive mapping may extend over multiple areas; and that few imaging studies have been performed at this age, we conducted an exploratory analysis examining all ROIs (Lyall et al., 2015). However, on the basis of prior literature, we hypothesised that areas of the frontal lobe would be associated with cognitive development, areas linked to language function would include frontal and temporal regions, and that there would be regions of shared cognitive and language function (Paterson et al., 2006). Age and sex were included as covariates in all analyses. Intracranial volume (ICV) was also added into the surface area analyses (Voevodskaya et al., 2014). We report standardised beta coefficients with 95% confidence intervals to illustrate effect sizes (Murner-Lavanchy et al., 2018; Nieminen et al., 2013). We focus on regions with an association with cognitive or language development with a beta coefficient ≥0.20 (a small-moderate effect size (Acock, 2014; Sawilowsky, 2009) and for illustrative purposes have constructed effect size maps with any regions with uncorrected p ​< ​0.05.

Additional analyses were performed including household income as a confounder given reports that socioeconomic status may affect the brain-cognition relationship (Brito et al., 2017). A sensitivity analysis was also performed excluding children who were classified as outliers (ENIGMA, 2019). All analyses were performed with STATA version 14.0.

### 2.9 Data and code availability statement

The de-identified data that support the findings of this study are available from the authors upon reasonable request as per cohort guidelines.

## 3 Results

**Fig. 1:**
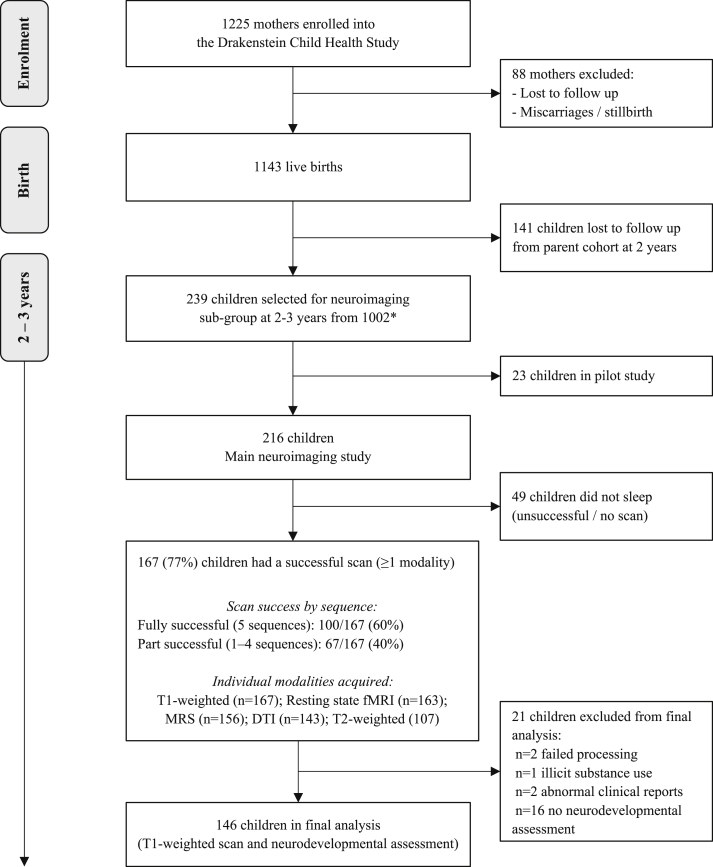
Flow chart for neuroimaging in the DCHS cohort and sequence success at 2–3 years. ∗Selection criteria: Fully described in section 2.3.2. Inclusion criteria: Currently active in the cohort, staying in the study area, child aged 2–3 years. Exclusion criteria: (i) Medical comorbidity (genetic syndrome, neurological disorder, or congenital abnormality); (ii) Gestation <36 weeks; (iii) Low Apgar score (<7 ​at 5 ​min); (iv) Neonatal intensive care admission; (v) Maternal use of illicit drugs during pregnancy; (vi) Child HIV infection.

A total of 239 children attended for imaging at 2–3 years; 23/239 (9.6%) for the pilot at Tygerberg CUBIC, and 216/239 (90.4%) for the main neuroimaging study to Groote Schuur CUBIC (Fig. 1).

Of those children attending the main neuroimaging study at Groote Schuur CUBIC (n ​= ​216), the median age was 34.8 months (IQR 33.7–35.6) and 122 (56%) were male. Children in the neuroimaging sub-group were representative of the full cohort at birth (n ​= ​1143), and those in follow up at 2 years (n ​= ​1002), with similar maternal educational attainment and overall socioeconomic status. However, a greater proportion of children in the neuroimaging sub-group were in the middle income category (Supplementary Tables 1 and 2).

### 3.1 Scan success

Overall, 167/216 children (77%) successfully completed at least T1-weighted scanning (Fig. 1). 49/216 (23%) children did not sleep and we were unable to obtain imaging data for these children. Of the 167 children with at least one successful sequence, 100/167 (60%) slept through the full 5 sequences (T1-MEMPRAGE, resting state fMRI, MRS, DTI and T2-weighted sequences) and 67/167 (40%) slept through between 1 and 4 sequences. In addition to T1-weighted images, 163/167 (98%) children had resting state fMRI, 156/167 (93%) MRS, 143/167 (86%) DTI and 107/167 (64%) T2-weighted images. Unsuccessful scans were primarily due to children not falling asleep which was often compounded by minor illness, for example a cough. There were no associations found between child age, child sex or neurodevelopment and scan success (Table 2).

**Table 2:** Association of scan success with sociodemographic factors and developmental outcomes (n ​= ​216).

Variable	Fully successful (5 sequences) (n ​= ​100)	1-4 successful sequences (n ​= ​67)	Unsuccessful (0 sequences) (n ​= ​49)	p-value^a^
**Age (months), mean (SD)**	34.2 (2.0)	34.5 (1.7)	34.7 (1.8)	0.30
**Sex (male)**	60 (60.0%)	37 (55.2%)	25 (51.0%)	0.57
**Cognitive development**				
Composite score, mean (SD)	86.4 (9.3)	86.0 (10.1)	85.1 (8.8)	0.74
Developmental delay ​< ​-1SD, n (%)	35 (38.5%)	21 (35.6%)	25 (53.2%)	0.15
**Language development**				
Composite score, mean (SD)	83.8 (10.1)	85.8 (13.5)	83.4 (10.5)	0.50
Developmental delay ​< ​-1SD, n (%)	48 (56.5%)	25 (44.6%)	26 (56.5%)	0.33

a Footnote: 1-way ANOVA and Chi-square tests performed to compare the three scan success groups.

### 3.2 Cortical structure and neurocognitive development

146/167 (87.4%) children had an included T1-weighted scan and neurocognitive assessment data available for these analyses (Fig. 1). Children had a mean age of 34.0 months and 57.5% were male (see Table 3). Of note, the proportions of children with developmental performance ​< ​-1SD below high income country norms included 37.0% in the cognitive domain and 52.2% in language, which are representative of the developmental outcomes reported across the broader DCHS.

**Table 3:** Sociodemographic and neurodevelopmental characteristics of children with an included T1-weighted scan and neurocognitive assessment (n ​= ​146).

Variable	N (%) or Mean (SD)
**Age months**	34.0 (1.7)
**Sex (male)**	84 (57.5%)
**Monthly household income (ZAR)**	
< ​R1000 (<~$75)	48 (32.9%)
R1000-R5000 (~$75–375)	86 (58.9%)
>R5000 (>~$375)	12 (8.2%)
**Maternal education**	
Primary	7 (4.8%)
Secondary	91 (62.3%)
Completed secondary	40 (27.4%)
Any tertiary	8 (5.5%)
**Maternal employment status (employed)**	41 (28.1%)
**SES quartile**	
Lowest SES	29 (19.9%)
Low-mod SES	35 (24.0%)
Mod-high SES	45 (30.8%)
High SES	37 (25.3%)
**Cognitive development**	
Composite score, mean (SD)	86.4 (9.3)
Developmental delay ​< ​-1SD, n (%)	54 (37.0%)
**Language development**	
Composite score, mean (SD)	84.4 (11.5)
Developmental delay ​< ​-1SD, n (%)	72 (52.2%)

Footnote: Missing data: Language (n ​= ​8).

**Fig. 2:**
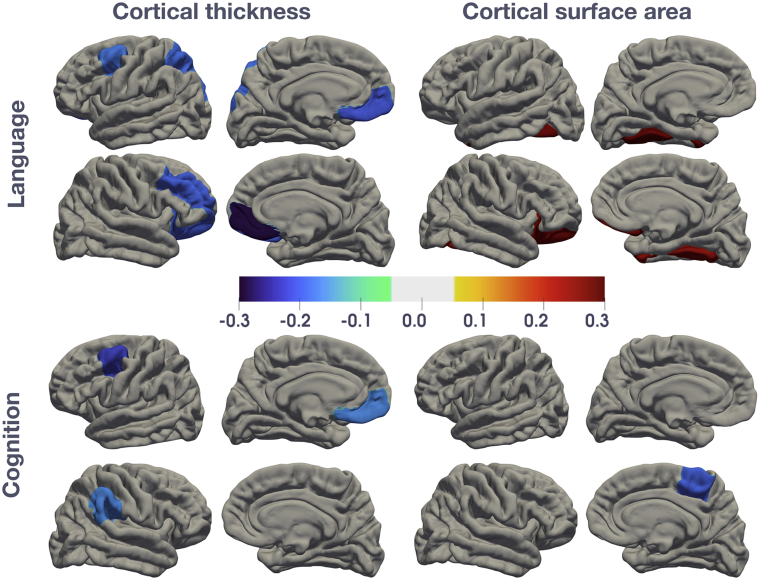
Statistical maps of effect size (beta coefficients) for the associations between cortical surface area and thickness with cognitive or language development. Beta coefficients are plotted for each region of interest on a template image. Standardised beta coefficients are calculated from multiple regression models adjusting for child age and sex, and additionally for intracranial volume for surface area measurements. Only significant (uncorrected p ​< ​0.05) regions are shown; non-significant regions are coloured in grey. Blue colours represent regions with negative beta coefficients and red represent positive beta coefficients. Please refer to Table 4 for more information.

Associations between cortical surface area and thickness and cognitive and language development are shown in Table 4 and Fig. 2. Cortical morphometry in the frontal lobe showed the strongest association with cognitive development including cortical surface area in the right paracentral region (beta coefficient −0.20 (95% confidence interval [CI] −0.39 to −0.01) and cortical thickness in the left caudal middle frontal region (beta coefficient −0.23 [95% CI −0.38 to −0.07]). Regions-of-interest in the frontal and temporal lobes were associated with language development. Cortical surface area in the left and right fusiform (beta coefficient 0.29 [95% CI 0.07 to 0.50] and 0.26 [95% CI 0.03 to 0.48] respectively), and right lateral orbitofrontal region (beta coefficient 0.27 [95% CI 0.03 to 0.52]) showed positive associations with language development. Overall, thinner cortices were associated with higher language scores. Cortical thickness in regions of the frontal lobe including the left and right medial orbitofrontal regions (beta coefficient −0.21 [95% CI −0.37 to −0.05] and −0.29 [95% CI −0.45 to −0.13] respectively), right lateral orbitofrontal (beta coefficient −0.20 (95% CI −0.35 to −0.04) and right rostral middle frontal areas (beta coefficient −0.20 [95% CI -0.36 to −0.04]) all showed negative associations with language with beta coefficients ≥0.20 (Table 4).

**Table 4:** Structural associations (cortical surface area and thickness) of regions-of-interest with cognitive or language development.

Cortical Surface Area	Lobe	Hemisphere	Mean	SD	Cognitive development (n ​= ​146)		Language development (n ​= ​138)	

Beta coefficient (95% CI)	*P*	Beta coefficient (95% CI)	*P*

Fusiform	Temporal	L	2660	335	0.04 (−0.18 to 0.26)	0.725	0.29 (0.07 to 0.50)∗∗	0.009∗
		R	2648	356	0.12 (−0.10 to 0.34)	0.269	0.26 (0.03 to 0.48)∗∗	0.024∗
Insula	Temporal	L	1992	204	−0.05 (−0.28 to 0.18)	0.693	0.09 (−0.15 to 0.33)	0.448
		R	1962	241	0.09 (−0.11 to 0.29)	0.393	0.20 (−0.00 to 0.41)∗∗	0.050
Lateral orbitofrontal	Frontal	L	2100	311	0.12 (−0.13 to 0.36)	0.346	0.22 (−0.03 to 0.47)∗∗	0.079
		R	2058	310	0.09 (−0.16 to 0.33)	0.493	0.27 (0.03 to 0.52)∗∗	0.028∗
Paracentral	Frontal	L	1192	169	−0.04 (−0.24 to 0.16)	0.699	0.11 (−0.09 to 0.30)	0.294
		R	1315	183	−0.20 (−0.39 to −0.01)∗∗	0.036∗	−0.12 (−0.31 to 0.07)	0.215
Cortical Thickness	Lobe	Hemisphere	Mean	SD	Beta coefficient (95% CI)	*P*	Beta coefficient (95% CI)	*P*
Caudal middle frontal	Frontal	L	2.98	0.17	−0.23 (−0.38 to −0.07)∗∗	0.006∗	−0.18 (−0.34 to −0.02)	0.027∗
		R	2.92	0.18	−0.13 (−0.29 to 0.03)	0.118	−0.11 (−0.28 to 0.05)	0.166
Lateral orbitofrontal	Frontal	L	3.32	0.17	−0.00 (−0.17 to 0.16)	0.956	−0.01 (−0.17 to 0.15)	0.918
		R	3.21	0.17	−0.11 (−0.27 to 0.05)	0.164	−0.20 (−0.35 to −0.04)∗∗	0.014∗
Medial orbitofrontal	Frontal	L	3.17	0.21	−0.17 (−0.33 to −0.01)	0.036∗	−0.21 (−0.37 to −0.05)∗∗	0.011∗
		R	3.18	0.23	−0.16 (−0.32 to 0.01)	0.057	−0.29 (−0.45 to −0.13)∗∗	0.001∗
Rostral middle frontal	Frontal	L	3.01	0.13	−0.14 (−0.30 to 0.02)	0.087	−0.10 (−0.26 to 0.06)	0.225
		R	2.91	0.13	−0.12 (−0.28 to 0.04)	0.136	−0.20 (−0.36 to −0.04)∗∗	0.016∗
Superior parietal	Parietal	L	2.63	0.13	−0.11 (−0.27 to 0.05)	0.171	−0.19 (−0.35 to −0.03)	0.019∗
		R	2.61	0.12	0.03 (−0.14 to 0.20)	0.701	−0.09 (−0.26 to 0.08)	0.318
Supramarginal	Parietal	L	3.03	0.13	−0.00 (−0.16 to 0.16)	0.992	−0.03 (−0.20 to 0.13)	0.696
		R	2.99	0.16	−0.17 (−0.33 to −0.01)	0.035∗	−0.15 (−0.31 to 0.01)	0.072

Footnote: Table showing the structural associations between cortical surface area and cortical thickness with cognitive or language development in regions of interest, if either hemisphere had an uncorrected p ​< ​0.05. All linear regression models included child age and sex as covariates; associations with surface area also included intracranial volume. The beta (standardised) regression coefficient represents the effect size or expected change in cognitive or language development (in standard deviations) with a one unit standard deviation change in the region-of-interest. Beta coefficients are reported to 2 decimal places. ∗p ​< ​0.05, ∗∗absolute beta coefficient≥0.20.

Given the differences between the cohort and neuroimaging sub-group in terms of household income, a further analyses was performed including household income as a confounder (Supplementary Table 3). We show that the effect estimates hold. A sensitivity analysis was also performed excluding children classified as extreme outliers (Supplementary Table 4) which did not substantially change the results.

## 4 Discussion

Our study demonstrates the feasibility of paediatric multimodal neuroimaging without sedation or anesthesia at 2–3 years in a sub-Saharan African setting, where 66% of children under-5 are at risk of developmental impairment (Black et al., 2017). We also provide preliminary analyses addressing relationships between cortical thickness and surface area with cognitive and language development in this age group. To our knowledge this is the first cohort study in SSA to report the association of neuroimaging with developmental data at this age. The methods used to encourage sleep are low-cost, associated with minimal risk and may be implemented in other LMIC settings, offering a viable alternative to sedation or anesthesia. Given the global focus on early child development (Black et al., 2017; Every Woman Every Child, 2015; McDonald et al., 2016) and the value of MRI in the investigation of brain development, this longitudinal cohort of in-depth structural and functional MRI may be useful in understanding the various factors affecting child brain development in this setting, so complementing work in high income countries (Almli et al., 2007; Walker et al., 2016).

Neuroimaging children has intrinsic challenges due to motion and limited cooperation, particularly in the preschool years (Barkovich et al., 2019). We showed a 77% success rate for scanning children without sedation. Neuroimaging young children during non-sedated sleep has not previously been described in a SSA setting, and our results compare favourably to studies in high income countries (Dean et al., 2014). A recent study of children in Canada aged 2–5 years showed success rates of 72% utilising a mock scanner to prepare for the MRI (Thieba et al., 2018), and a large US study reported two thirds success in children between 0 and 5 years (Almli et al., 2007). Imaging children under the age of 5 years without sedation is logistically challenging and it was not developmentally appropriate to use a mock scanner or audio/visual systems emulating MRI sounds in children under 3 years. However, sedation and anesthesia have safety and ethical limitations (Schmidt et al., 2011), utilise resources, and may not be used on all children (including those with respiratory issues). Furthermore, there have been reports of later developmental and behavioural risks associated with anesthesia exposure in childhood (DiMaggio et al., 2011). We conducted MRI during natural sleep adapting methods that have been shown to be successful in high income countries (Raschle et al., 2012; Jaimes and Gee, 2016) to this setting. Our dedicated team used behavioural and play therapy techniques to maximise the parent and child’s comfort and minimise distress, with optimisation of the scanner environment to create a child-friendly space conducive to sleeping. Imaging times were coordinated with child sleeping and nap times. We used melatonin, a neurohormone recognised for its regulation of sleep via the circadian rhythm (Abdelgadir et al., 2018) which has been shown to be safe and effective for inducing sleep in children in our setting without the risk of respiratory compromise or the requirement of specialist monitoring (Ibekwe et al., 2017; Johnson et al., 2002).

Overall, we have shown that imaging during natural sleep is a feasible alternative to sedation that can result in minimal motion and high quality scans, addressing the importance of scanning in LMIC settings (Maxfield et al., 2019). We found scan success was not associated with child age, sex or neurodevelopmental scores suggesting natural sleep may be used without resulting in selection bias. These approaches may be used to allow effective imaging without sedation in future studies. Furthermore, although this work was conducted in a research context, it has important implications for clinical settings in terms of reducing the number of children needing sedation and anesthesia in routine clinical investigations. In addition, MRI may be performed in locations with limited monitoring and resuscitation facilities (Johnson et al., 2002).

In this study we also explored whether regional variations in brain structure were associated with neurocognitive function at 2–3 years of age. Whereas there are well-defined regional differences in cortical development trajectories in older children, adolescents and adults, very little is known about cortical maturation at this age, particularly in LMIC (Gilmore et al., 2012; Deoni et al., 2015). We report on both cortical surface area and thickness, and found that cognitive development was most strongly associated with cortical morphometry in frontal regions. This is consistent with studies across all ages where well-defined associations between cognition and frontal areas are reported (Paterson et al., 2006; Shaw et al., 2006; Narr et al., 2007). Studies of older children have identified the frontal lobes, and maturation of the prefrontal cortex in particular, as important for later cognitive tasks, including executive function (Paterson et al., 2006; Ronan et al., 2019). Notably, the frontal areas identified are major heteromodal cortical association regions, suggesting their structural maturation is key to integrated higher-level function.

Language development was positively associated with cortical surface area in temporal and frontal regions, and negatively associated with cortical thickness in multiple frontal areas. Our regional findings are consistent with data from functional studies of younger children which show language associated with activation of the temporal lobe as well as frontal regions (Imada et al., 2006; Dehaene-Lambertz et al., 2002). In adults, there is evidence for a key role of the temporal region (specifically the fusiform gyrus) as well as collateral recruitment of frontal cortex in language (Paterson et al., 2006; Sowell et al., 2003). Other studies have also found cortical thickness negatively correlated to language (and executive function) in childhood, predominantly in the frontal and temporal cortical regions (Shaw et al., 2006; Murner-Lavanchy et al., 2018; Brito et al., 2017; Girault et al., 2019; Porter et al., 2011) Separately, white matter in frontal and temporal cortices has also been linked to receptive and expressive language development (O’Muircheartaigh et al., 2014). The orbitofrontal region showed associations with language for both cortical surface area and thickness. This area of the prefrontal cortex has been identified as important for higher functions later in life, including language-related tasks such as sentence completion (Elliott et al., 2000), and the relationship with language at this age may lay the groundwork for later higher order functions and more complex language development. Our results show associations in bilateral hemispheres consistent with findings from a study measuring white matter and language (Walton et al., 2018), which suggested that preschool children may use an extensive language processing network that becomes more left lateralised from 5 years (Paterson et al., 2006; Weiss-Croft and Baldeweg, 2015; Bates et al., 1997). Overall these exploratory analyses support current evidence of a dynamic interplay between brain structure and function over the first few years of life.

Cortical thickness and surface area are key components of cortical structure and their maturation over the first years is thought to play a critical role in later developmental outcomes (Remer et al., 2017). There are few previous studies of cortical surface area and thickness in this age group. In this study higher neurocognitive development scores were associated with reduced cortical thickness and generally increased surface area in specific regions. The negative associations of cognitive and language development with cortical thickness are consistent with previous reports (Shaw et al., 2006). Cortical thickness increases in infancy until two years (Lyall et al., 2015; Wang et al., 2019) followed by region-specific cortical thinning thereafter (Remer et al., 2017). The decreases in cortical thickness may reflect synaptic pruning or increased myelination (Brito et al., 2017; Natu et al., 2019), and the creation of a more efficient cortical network. Decreases in cortical thickness have been associated with improved cognitive performance, particularly in the frontal regions (Murner-Lavanchy et al., 2018; Wang et al., 2019; Burgaleta et al., 2014). A recent study reported cortical thickness decreases from as early as 1 year in some regions (Remer et al., 2017). Cortical surface area increases at a slower rate than cortical thickness and peaks later around nine years (Wierenga et al., 2018; Gilmore et al., 2018) consistent with the associations we found at aged 2–3 years being mainly positive. Potentially one explanation for the different direction of association in the cognitive analysis may be areas peaking earlier in development.

There are methodological considerations associated with this type of imaging research in young children. Incidental findings need to be managed responsibly (Jansen et al., 2017). We only report two incidental findings of clinical significance and the appropriate referral pathways were in place. In the structure-cognition analyses, overall, effects were modest but similar in magnitude to other studies examining brain structure and cognitive function (Gilmore et al., 2018; Shaw et al., 2006; Narr et al., 2007; Ronan et al., 2019) Longitudinal studies and larger sample sizes are likely needed to show strong associations given the extensive brain development over the first few years of life and differences in individual brain trajectories. One study also found that the dynamic change in cortical structure was more closely related to intelligence than a static measure (Shaw et al., 2006). Furthermore, as multiple regions are responsible for neurocognitive function, investigating other modalities, including diffusion tensor imaging and functional MRI, will be useful to inform the network of regions and connections at the brain-cognition interface.

This is the first study to investigate both cortical structure and thickness in preschool South African children and the relationship with cognitive function. Strengths of this study include the comprehensive neuroimaging and clinical assessments and large sample size. This study also has limitations. The neuroimaging sub-group inclusion criteria were based upon the data collected up to the point of sample selection, and the accuracy of gestational age data was limited by the tools available. Secondly, the results are exploratory and do not allow causal inference. We have reported uncorrected p-values throughout due to the approach of this analysis and therefore our results must be interpreted as exploratory and require replication in other studies. We therefore focus on effect sizes throughout and discuss the strength of associations. Thirdly, we adjusted for child age and sex but not for other potential sociodemographic confounding factors in this proof-of-concept analysis, and further work is needed to explore these. Overall, our sample was representative of the wider DCHS parent study in terms of sociodemographic variables, differing only in household income which did not substantially affect results in our sensitivity analyses and there is little variability across the DCHS. Brito and colleagues found socioeconomic disadvantage may exaggerate links between brain structure and cognitive development (Brito et al., 2017). In that study, negative associations between cortical thickness and cognition were more robust for children from lower socioeconomic status homes, similar to the majority of families in the DCHS cohort. We therefore feel the results are generalisable to the wider population.

From a public health perspective, given the global burden of neurodevelopmental impairment in children is greatest in LMIC, neuroimaging research in these contexts is necessary to map brain development, cognition and early origins of disease (Dubois et al., 2014; Morita et al., 2016). Overall, these findings are consistent with our *a priori* hypotheses that frontal regions show (unadjusted within the selected ROIs) associations of thickness and surface area with cognitive development, and frontal and temporal areas with language. This lays the foundation for further work to explore the genetic and environmental influences on child development (Gilmore et al., 2018). Future hypotheses-driven analyses will examine the impact of environmental factors on brain structure and function in the DCHS cohort (Donald et al., 2015; Tran et al., 2016).

## 5 Conclusions

In conclusion, we demonstrate a successful methodological approach to neuroimaging without sedation under the age of 5 years in sub-Saharan Africa. Given the importance of early child development and use of MRI to investigate developmental delay, further studies may utilise these approaches to allow effective imaging during natural sleep. In this proof-of-concept analysis we also demonstrate regional associations between cortical surface area and thickness with cognitive and language development at 2–3 years, similar to brain-cognition studies from high income settings. These provide future directions for understanding early child development and to examine the impact of socioenvironmental factors in this context.

## Funding

The DCHS study is funded by the Bill and Melinda Gates Foundation [OPP 1017641]. Additional support for HJZ and DJS by the Medical Research Council of South Africa. CJW is supported by the Wellcome Trust through a Research Training Fellowship [203525/Z/16/Z]. KAD and aspects of the research are additionally supported by the NRF, an Academy of Medical Sciences Newton Advanced Fellowship (NAF002/1001) funded by the UK Government’s Newton Fund, by NIAAA via (R21AA023887), by the Collaborative Initiative on Fetal Alcohol Spectrum Disorders (CIFASD) developmental grant (U24 AA014811), and by the US Brain and Behaviour Foundation Independent Investigator grant (24467). AMR is additionally supported by the UK Medical Research Council (MRC) and the UK Department for International Development (DFID) under the MRC/DFID Concordat agreement which is also part of the EDCTP2 programme supported by the European Union grant reference (MR/R010161/1).

The funders had no role in the study design, data collection, analysis or interpretation, report writing or decision to submit for publication. The corresponding author had full access to study data and final responsibility for the decision to submit for publication.

## CRediT authorship contribution statement

**Catherine J. Wedderburn:** Conceptualization, Methodology, Investigation, Formal analysis, Visualization, Writing - original draft, Writing - review & editing. **Sivenesi Subramoney:** Data curation, Investigation, Writing - review & editing. **Shunmay Yeung:** Supervision, Writing - review & editing. **Jean-Paul Fouche:** Software, Formal analysis, Writing - review & editing. **Shantanu H. Joshi:** Software, Visualization, Writing - review & editing. **Katherine L. Narr:** Conceptualization, Methodology, Resources, Writing - review & editing. **Andrea M. Rehman:** Formal analysis, Writing - review & editing. **Annerine Roos:** Investigation, Writing - review & editing. **Jonathan Ipser:** Methodology, Software, Validation. **Frances C. Robertson:** Resources, Writing - review & editing. **Nynke A. Groenewold:** Validation, Writing - review & editing. **Diana M. Gibb:** Supervision, Writing - review & editing. **Heather J. Zar:** Conceptualization, Methodology, Resources, Writing - review & editing. **Dan J. Stein:** Conceptualization, Methodology, Resources, Writing - review & editing. **Kirsten A. Donald:** Conceptualization, Methodology, Resources, Investigation, Supervision, Writing - review & editing.

## Declaration of competing interest

The authors report no conflicts of interest.

## References

[bib1] Abdelgadir I.S., Gordon M.A., Akobeng A.K. (2018). Melatonin for the management of sleep problems in children with neurodevelopmental disorders: a systematic review and meta-analysis. Arch. Dis. Child..

[bib2] Acock A.C. (2014). A Gentle Introduction to Stata.

[bib3] Albers C.A., Grieve A.J. (2007). Review of Bayley scales of infant and toddler development--third edition. J. Psychoeduc. Assess..

[bib4] Almli C.R., Rivkin M.J., McKinstry R.C. (2007). Brain Development Cooperative G. The NIH MRI study of normal brain development (Objective-2): newborns, infants, toddlers, and preschoolers. Neuroimage.

[bib5] Azhari A., Truzzi A., Neoh M.J. (2019). A decade of infant neuroimaging research: what have we learned and where are we going?. Infant Behav. Dev..

[bib6] Ballot D.E., Potterton J., Chirwa T., Hilburn N., Cooper P.A. (2012). Developmental outcome of very low birth weight infants in a developing country. BMC Pediatr..

[bib7] Barkovich M.J., Li Y., Desikan R.S., Barkovich A.J., Xu D. (2019). Challenges in pediatric neuroimaging. Neuroimage.

[bib8] Bates E., Broman S., Fletcher J. (1997). Plasticity, localization and language development. The Changing Nervous System: Neurobehavioral Consequences of Early Brain Disorders.

[bib9] Bayley N. (2006). Bayley Scales of Infant and Toddler Development, Technical Manual.

[bib10] Black M.M., Walker S.P., Fernald L.C.H. (2017). Early childhood development coming of age: science through the life course. Lancet.

[bib11] Brito N.H., Piccolo L.R., Noble K.G. (2017). Pediatric Imaging N, Genetics S. Associations between cortical thickness and neurocognitive skills during childhood vary by family socioeconomic factors. Brain Cognit..

[bib12] Burgaleta M., Johnson W., Waber D.P., Colom R., Karama S. (2014). Cognitive ability changes and dynamics of cortical thickness development in healthy children and adolescents. Neuroimage.

[bib14] Cote C.J., Notterman D.A., Karl H.W., Weinberg J.A., McCloskey C. (2000). Adverse sedation events in pediatrics: a critical incident analysis of contributing factors. Pediatrics.

[bib15] Daelmans B., Darmstadt G.L., Lombardi J. (2017). Early childhood development: the foundation of sustainable development. Lancet.

[bib16] Dale A.M., Fischl B., Sereno M.I. (1999). Cortical surface-based analysis. I. Segmentation and surface reconstruction. Neuroimage.

[bib17] Dean D.C., Dirks H., O’Muircheartaigh J. (2014). Pediatric neuroimaging using magnetic resonance imaging during non-sedated sleep. Pediatr. Radiol..

[bib18] Dehaene-Lambertz G., Dehaene S., Hertz-Pannier L. (2002). Functional neuroimaging of speech perception in infants. Science.

[bib19] Dehaene-Lambertz G., Montavont A., Jobert A. (2010). Language or music, mother or Mozart? Structural and environmental influences on infants’ language networks. Brain Lang..

[bib20] Deoni S.C., Dean D.C., Remer J., Dirks H., O’Muircheartaigh J. (2015). Cortical maturation and myelination in healthy toddlers and young children. Neuroimage.

[bib21] Desikan R.S., Segonne F., Fischl B. (2006). An automated labeling system for subdividing the human cerebral cortex on MRI scans into gyral based regions of interest. Neuroimage.

[bib22] DiMaggio C., Sun L.S., Li G. (2011). Early childhood exposure to anesthesia and risk of developmental and behavioral disorders in a sibling birth cohort. Anesth. Analg..

[bib23] Donald K.A., Roos A., Fouche J.P. (2015). A study of the effects of prenatal alcohol exposure on white matter microstructural integrity at birth. Acta Neuropsychiatr..

[bib24] Donald K.A., Hoogenhout M., du Plooy C.P. (2018). Drakenstein Child Health Study (DCHS): investigating determinants of early child development and cognition. BMJ Paediatr. Open.

[bib25] Dubois J., Dehaene-Lambertz G., Kulikova S., Poupon C., Huppi P.S., Hertz-Pannier L. (2014). The early development of brain white matter: a review of imaging studies in fetuses, newborns and infants. Neuroscience.

[bib26] Edwards A.D., Arthurs O.J. (2011). Paediatric MRI under sedation: is it necessary? What is the evidence for the alternatives?. Pediatr. Radiol..

[bib27] Elliott R., Dolan R.J., Frith C.D. (2000). Dissociable functions in the medial and lateral orbitofrontal cortex: evidence from human neuroimaging studies. Cerebr. Cortex.

[bib28] ENIGMA Structural image processing protocols. http://enigma.ini.usc.edu/protocols/imaging-protocols/.

[bib37] Every Woman Every Child (2015). The Global Strategy for Women’s, Children’s and Adolescents’ Health (2016-2030). http://www.who.int/pmnch/activities/advocacy/globalstrategy/2016_2030/en/.

[bib29] Fischl B., Dale A.M. (2000). Measuring the thickness of the human cerebral cortex from magnetic resonance images. Proc. Natl. Acad. Sci. U. S. A..

[bib30] Fischl B., Salat D.H., Busa E. (2002). Whole brain segmentation: automated labeling of neuroanatomical structures in the human brain. Neuron.

[bib31] Fischl B., Salat D.H., van der Kouwe A.J. (2004). Sequence-independent segmentation of magnetic resonance images. Neuroimage.

[bib32] Gao W., Grewen K., Knickmeyer R.C. (2019). A review on neuroimaging studies of genetic and environmental influences on early brain development. Neuroimage.

[bib33] Gilmore J.H., Shi F., Woolson S.L. (2012). Longitudinal development of cortical and subcortical gray matter from birth to 2 years. Cerebr. Cortex.

[bib34] Gilmore J.H., Knickmeyer R.C., Gao W. (2018). Imaging structural and functional brain development in early childhood. Nat. Rev. Neurosci..

[bib35] Girault J.B., Cornea E., Goldman B.D. (2019). Cortical structure and cognition in infants and toddlers. Cerebr. Cortex.

[bib36] Hagler D.J., Hatton S., Cornejo M.D. (2019). Image processing and analysis methods for the adolescent brain cognitive development study. Neuroimage.

[bib38] Hermoye L., Saint-Martin C., Cosnard G. (2006). Pediatric diffusion tensor imaging: normal database and observation of the white matter maturation in early childhood. Neuroimage.

[bib39] Ibekwe R., Jeaven L., Wilmshurst J.M. (2017). The role of melatonin to attain electroencephalograms in children in a sub-Saharan African setting. Seizure.

[bib40] Imada T., Zhang Y., Cheour M., Taulu S., Ahonen A., Kuhl P.K. (2006). Infant speech perception activates Broca’s area: a developmental magnetoencephalography study. Neuroreport.

[bib41] Jahanshad N., Couture M.C., Prasitsuebsai W. (2015). Brain imaging and neurodevelopment in HIV-uninfected Thai children born to HIV-infected mothers. Pediatr. Infect. Dis. J..

[bib42] Jaimes C., Gee M.S. (2016). Strategies to minimize sedation in pediatric body magnetic resonance imaging. Pediatr. Radiol..

[bib43] Jansen P.R., Dremmen M., van den Berg A. (2017). Incidental findings on brain imaging in the general pediatric population. N. Engl. J. Med..

[bib44] Jevtovic-Todorovic V., Absalom A.R., Blomgren K. (2013). Anaesthetic neurotoxicity and neuroplasticity: an expert group report and statement based on the BJA Salzburg Seminar. Br. J. Anaesth..

[bib45] Johnson K., Page A., Williams H., Wassemer E., Whitehouse W. (2002). The use of melatonin as an alternative to sedation in uncooperative children undergoing an MRI examination. Clin. Radiol..

[bib46] Knickmeyer R.C., Gouttard S., Kang C. (2008). A structural MRI study of human brain development from birth to 2 years. J. Neurosci..

[bib47] Lyall A.E., Shi F., Geng X. (2015). Dynamic development of regional cortical thickness and surface area in early childhood. Cerebr. Cortex.

[bib48] Maxfield C.M., Haberle S., Nijssen-Jordan C., Mollura D.J., Culp M.P., Lungren M.P. (2019). Pediatric imaging in global health radiology. Radiology in Global Health: Strategies, Implementation, and Applications.

[bib49] McDonald S., Kehler H., Bayrampour H., Fraser-Lee N., Tough S. (2016). Risk and protective factors in early child development: results from the All Our Babies (AOB) pregnancy cohort. Res. Dev. Disabil..

[bib50] Morita T., Asada M., Naito E. (2016). Contribution of neuroimaging studies to understanding development of human cognitive brain functions. Front. Hum. Neurosci..

[bib51] Murner-Lavanchy I., Rummel C., Steinlin M., Everts R. (2018). Cortical morphometry and cognition in very preterm and term-born children at early school age. Early Hum. Dev..

[bib52] Myer L., Stein D.J., Grimsrud A., Seedat S., Williams D.R. (2008). Social determinants of psychological distress in a nationally-representative sample of South African adults. Soc. Sci. Med..

[bib53] Narr K.L., Woods R.P., Thompson P.M. (2007). Relationships between IQ and regional cortical gray matter thickness in healthy adults. Cerebr. Cortex.

[bib54] Natu V.S., Gomez J., Barnett M. (2019). Apparent thinning of human visual cortex during childhood is associated with myelination. Proc. Natl. Acad. Sci. U. S. A..

[bib55] Nieminen P., Lehtiniemi H., Vähäkangas K., Huusko A., Rautio A. (2013). Standardised regression coefficient as an effect size index in summarising findings in epidemiological studies. Epediamiol. Biostat. Publ. Health.

[bib56] Nwosu E.C., Robertson F.C., Holmes M.J. (2018). Altered brain morphometry in 7-year old HIV-infected children on early ART. Metab. Brain Dis..

[bib57] O’Muircheartaigh J., Dean D.C., Ginestet C.E. (2014). White matter development and early cognition in babies and toddlers. Hum. Brain Mapp..

[bib58] Ouyang M., Dubois J., Yu Q., Mukherjee P., Huang H. (2019). Delineation of early brain development from fetuses to infants with diffusion MRI and beyond. Neuroimage.

[bib13] Paediatric Formulary Committee (2016-2017). BNF for Children. http://www.bnf.org.

[bib59] Paterson S.J., Heim S., Friedman J.T., Choudhury N., Benasich A.A. (2006). Development of structure and function in the infant brain: implications for cognition, language and social behaviour. Neurosci. Biobehav. Rev..

[bib60] Porter J.N., Collins P.F., Muetzel R.L., Lim K.O., Luciana M. (2011). Associations between cortical thickness and verbal fluency in childhood, adolescence, and young adulthood. Neuroimage.

[bib61] Rademeyer V., Jacklin L. (2013). A study to evaluate the performance of black South African urban infants on the Bayley Scales of Infant Development III. S. Afr. J. Child Health.

[bib62] Raschle N., Zuk J., Ortiz-Mantilla S. (2012). Pediatric neuroimaging in early childhood and infancy: challenges and practical guidelines. Ann. N. Y. Acad. Sci..

[bib63] Remer J., Croteau-Chonka E., Dean D.C. (2017). Quantifying cortical development in typically developing toddlers and young children, 1-6 years of age. Neuroimage.

[bib64] Ronan L., Alexander-Bloch A., Fletcher P.C. (2019). Childhood obesity, cortical structure, and executive function in healthy children. Cerebr. Cortex.

[bib65] Sammons H.M., Edwards J., Rushby R., Picton C., Collier J., Whitehouse W.P. (2011). General anaesthesia or sedation for paediatric neuroimaging: current practice in a teaching hospital. Arch. Dis. Child..

[bib66] Sawilowsky S.S. (2009). New effect size rules of thumb. J. Mod. Appl. Stat. Methods.

[bib67] Schmidt M.H., Marshall J., Downie J., Hadskis M.R. (2011). Pediatric magnetic resonance research and the minimal-risk standard. IRB.

[bib68] Shaw P., Greenstein D., Lerch J. (2006). Intellectual ability and cortical development in children and adolescents. Nature.

[bib69] Sowell E.R., Peterson B.S., Thompson P.M., Welcome S.E., Henkenius A.L., Toga A.W. (2003). Mapping cortical change across the human life span. Nat. Neurosci..

[bib70] Stats SA (2016). Statistical Release: Recorded Live Births 2013-2015.

[bib71] Stein D.J., Koen N., Donald K.A. (2015). Investigating the psychosocial determinants of child health in Africa: the Drakenstein child health study. J. Neurosci. Methods.

[bib72] Thieba C., Frayne A., Walton M. (2018). Factors associated with successful MRI scanning in unsedated young children. Front. Pediatr..

[bib73] Tomlinson M., Bornstein M.H., Marlow M., Swartz L. (2014). Imbalances in the knowledge about infant mental health in rich and poor countries: too little progress in bridging the gap. Infant Ment. Health J..

[bib74] Tran L.T., Roos A., Fouche J.P. (2016). White matter microstructural integrity and neurobehavioral outcome of HIV-exposed uninfected neonates. Medicine (Baltim.).

[bib75] Voevodskaya O., Simmons A., Nordenskjold R. (2014). The effects of intracranial volume adjustment approaches on multiple regional MRI volumes in healthy aging and Alzheimer’s disease. Front. Aging Neurosci..

[bib76] Walker S.P., Wachs T.D., Grantham-McGregor S. (2011). Inequality in early childhood: risk and protective factors for early child development. Lancet.

[bib77] Walker L., Chang L.C., Nayak A. (2016). The diffusion tensor imaging (DTI) component of the NIH MRI study of normal brain development (PedsDTI). Neuroimage.

[bib78] Walton M., Dewey D., Lebel C. (2018). Brain white matter structure and language ability in preschool-aged children. Brain Lang..

[bib79] Wang F., Lian C., Wu Z. (2019). Developmental topography of cortical thickness during infancy. Proc. Natl. Acad. Sci. U. S. A..

[bib80] Weiss-Croft L.J., Baldeweg T. (2015). Maturation of language networks in children: a systematic review of 22years of functional MRI. Neuroimage.

[bib81] Wierenga L.M., van den Heuvel M.P., Oranje B. (2018). A multisample study of longitudinal changes in brain network architecture in 4-13-year-old children. Hum. Brain Mapp..

[bib82] Zar H.J., Barnett W., Myer L., Stein D.J., Nicol M.P. (2015). Investigating the early-life determinants of illness in Africa: the Drakenstein child health study. Thorax.

