# Supplementary material for: Neuroimaging young children and associations with neurocognitive development in a South African birth cohort study
Source: Neuroimage. 2020 Oct 1;219:116846. doi: 10.1016/j.neuroimage.2020.116846 (PMC7443699; doi:10.1016/j.neuroimage.2020.116846)
Supplement: Multimedia component 1 [file mmc1.pdf]

Supplementary Material

Supplementary data for Wedderburn *et al.*, Neuroimaging young children and associations with neurocognitive development in a South African birth cohort study.

Appendix Table 1: Comparison of socioeconomic variables among children in the neuroimaging sub-group and the full birth cohort (n=1143)

Variables	Final analysis group (n=146)			Full imaging cohort (n=239)		
	Imaging (n=146)	No Imaging (n=997)	***P***	Imaging (n=239)	No Imaging (n=904)	***P***
Male sex	84 (57.5%)	504 (50.6%)	0.115	135 (56.5%)	453 (50.1%)	0.079
Monthly household income (ZAR)						
< R1000 (<~$75)	48 (32.9%)	383 (38.4%)		78 (32.6%)	353 (39.1%)	
R1000-R5000 (~$75-375)	86 (58.9%)	471 (47.2%)		139 (58.2%)	418 (46.2%)	
>R5000 (>~$375)	12 (8.2%)	143 (14.3%)	0.017*	22 (9.2%)	133 (14.7%)	0.003*
Maternal education						
Primary	7 (4.8%)	79 (7.9%)		14 (5.9%)	72 (8.0%)	
Secondary	91 (62.3%)	518 (52.0%)		143 (59.8%)	466 (51.6%)	
Completed secondary	40 (27.4%)	335 (33.6%)		70 (29.3%)	305 (33.7%)	
Any tertiary	8 (5.5%)	65 (6.5%)	0.115	12 (5.0%)	61 (6.8%)	0.134
Maternal employment status (employed)	41 (28.1%)	266 (26.7%)	0.721	67 (28.0%)	240 (26.6%)	0.645
SES quartile						
Lowest SES	29 (19.9%)	245 (24.6%)		46 (19.3%)	228 (25.2%)	
Low-mod SES	35 (24.0%)	261 (26.2%)		66 (27.6%)	230 (25.4%)	
Mod-high SES	45 (30.8%)	245 (24.6%)		69 (28.9%)	221 (24.5%)	
High SES	37 (25.3%)	246 (24.7%)	0.334	58 (24.3%)	225 (24.9%)	0.200

Footnotes:
*p<0.05; Chi-squared for categorical variables (n and % proportions presented).

Appendix Table 2: Comparison of socioeconomic variables among children in the neuroimaging sub-group and those in DCHS follow up at 2 years (n=1002)

**Variables**	**Imaging & Neurodevelopment (n=146)**			**Full imaging cohort (n=239)**		
	**Imaging (n=146)**	**No Imaging (n=856)**	***P***	**Imaging (n=239)**	**No Imaging (n=763)**	***P***
Male sex	84 (57.5%)	429 (50.1%)	0.097	135 (56.5%)	378 (49.5%)	0.061
Monthly household income (ZAR)						
< R1000 (<~$75)	48 (32.9%)	338 (39.5%)		78 (32.6%)	308 (40.4%)	
R1000-R5000 (~$75-375)	86 (58.9%)	406 (47.4%)		139 (58.2%)	353 (46.3%)	
>R5000 (>~$375)	12 (8.2%)	112 (13.15)	0.028*	22 (9.2%)	102 (13.4%)	0.005*
Maternal education						
Primary	7 (4.8%)	66 (7.7%)		14 (5.9%)	59 (7.7%)	
Secondary	91 (62.3%)	458 (53.5%)		143 (59.8%)	406 (53.2%)	
Completed secondary	40 (27.4%)	281 (32.8%)		70 (29.3%)	251 (32.9%)	
Any tertiary	8 (5.5%)	51 (6.0%)	0.219	12 (5.0%)	47 (6.2%)	0.324
Maternal employment status (employed)	41 (28.1%)	223 (26.1%)	0.607	67 (28.0%)	197 (25.8%)	0.498
SES quartile						
Lowest SES	29 (19.9%)	213 (24.9%)		46 (19.3%)	196 (25.7%)	
Low-mod SES	35 (24.0%)	233 (27.2%)		66 (27.6%)	202 (26.5%)	
Mod-high SES	45 (30.8%)	208 (24.3%)		69 (28.9%)	184 (24.1%)	
High SES	37 (25.3%)	202 (23.6%)	0.256	58 (24.3%)	181 (23.7%)	0.182

Footnotes:
*p<0.05; Chi-squared for categorical variables (n and % proportions presented).

Appendix Table 3: Associations of anatomical ROIs (cortical surface area and thickness) with either cognitive or language development adjusting for household income

			Cognitive development (n=146)		Language development (n=138)	
**Cortical Surface Area**	**Lobe**	**Hemisphere**	**Beta coefficient (95% CI)**	***P***	**Beta coefficient (95% CI)**	***P***
**Fusiform**	Temporal	L	0.04 (-0.17 to 0.26)	0.685	0.27 (0.06 to 0.49)**	0.013*
		R	0.15 (-0.06 to 0.37)	0.157	0.26 (0.04 to 0.48)**	0.023*
**Insula**	Temporal	L	-0.03 (-0.25 to 0.20)	0.815	0.09 (-0.14 to 0.33)	0.440
		R	0.09 (-0.11 to 0.28)	0.398	0.21 (0.01 to 0.41)**	0.043*
**Lateral orbitofrontal**	Frontal	L	0.13 (-0.11 to 0.37)	0.282	0.25 (0.00 to 0.49)**	0.048*
		R	0.10 (-0.14 to 0.34)	0.421	0.28 (0.04 to 0.52)**	0.024*
**Paracentral**	Frontal	L	-0.03 (-0.22 to 0.17)	0.785	0.12 (-0.08 to 0.32)	0.226
		R	-0.18 (-0.37 to 0.00)	0.051	-0.12 (-0.31 to 0.07)	0.216
**Cortical Thickness**	**Lobe**	**Hemisphere**	**Beta coefficient (95% CI)**	***P***	**Beta coefficient (95% CI)**	***P***
**Caudal middle frontal**	Frontal	L	-0.22 (-0.38 to -0.07)**	0.005*	-0.17 (-0.33 to -0.01)	0.034*
		R	-0.14 (-0.30 to 0.02)	0.075	-0.12 (-0.28 to 0.05)	0.158
**Lateral orbitofrontal**	Frontal	L	-0.02 (-0.18 to 0.14)	0.829	-0.01 (-0.17 to 0.15)	0.891
		R	-0.12 (-0.28 to 0.04)	0.126	-0.19 (-0.35 to -0.03)	0.017*
**Medial orbitofrontal**	Frontal	L	-0.19 (-0.35 to -0.03)	0.021*	-0.20 (-0.36 to -0.04)**	0.012*
		R	-0.18 (-0.34 to -0.02)	0.032*	-0.29 (-0.45 to -0.13)**	0.001*
**Rostral middle frontal**	Frontal	L	-0.17 (-0.33 to -0.01)	0.038*	-0.10 (-0.27 to 0.06)	0.225
		R	-0.14 (-0.30 to 0.02)	0.083	-0.20 (-0.36 to -0.04)**	0.018*
**Superior parietal**	Parietal	L	-0.15 (-0.32 to 0.01)	0.063	-0.22 (-0.38 to -0.06)**	0.009*
		R	0.02 (-0.15 to 0.19)	0.856	-0.08 (-0.26 to 0.09)	0.333
**Supramarginal**	Parietal	L	-0.04 (-0.20 to 0.12)	0.618	-0.05 (-0.22 to 0.12)	0.562
		R	-0.20 (-0.36 to -0.04)**	0.014*	-0.16 (-0.32 to 0.00)	0.056

Footnote

Table showing the ROIs with associations for cognitive and language development with cortical surface area and cortical thickness, if either hemisphere had p<0.05. All linear regression models included child age, child sex and household income as covariates; associations with surface area also included intracranial volume. The beta (standardised) regression coefficient represents the effect size or expected change in cognitive or language development (in standard deviations) with a one unit standard deviation change in the region-of-interest. Beta coefficients are reported to 2 decimal places. *p<0.05, **absolute beta coefficient>=0.20

Appendix Table 4: Associations of anatomical ROIs (cortical surface area and thickness) with either cognitive or language development (excluding outliers)

			Cognitive development (n=144)		Language development (n=136)	
**Cortical Surface Area**	**Lobe**	**Hemisphere**	**Beta coefficient (95% CI)**	***P***	**Beta coefficient (95% CI)**	***P***
**Fusiform**	Temporal	L	0.02 (-0.20 to 0.24)	0.862	0.28 (0.06 to 0.50)**	0.014*
		R‡	0.15 (-0.08 to 0.38)	0.199	0.28 (0.05 to 0.52)**	0.019*
**Insula**	Temporal	L	-0.05 (-0.28 to 0.18)	0.676	0.08 (-0.16 to 0.32)	0.503
		R	0.07 (-0.13 to 0.28)	0.486	0.19 (-0.01 to 0.40)	0.067
**Lateral orbitofrontal**	Frontal	L	0.12 (-0.13 to 0.36)	0.350	0.22 (-0.03 to 0.46)**	0.089
		R	0.08 (-0.17 to 0.33)	0.522	0.27 (0.02 to 0.52)**	0.032*
**Paracentral**	Frontal	L	-0.06 (-0.26 to 0.15)	0.579	0.11 (-0.10 to 0.31)	0.302
		R	-0.20 (-0.39 to -0.01)**	0.035*	-0.12 (-0.31 to 0.07)	0.221
**Cortical Thickness**	**Lobe**	**Hemisphere**	**Beta coefficient (95% CI)**	***P***	**Beta coefficient (95% CI)**	***P***
**Caudal middle frontal**	Frontal	L	-0.23 (-0.39 to -0.07)**	0.006*	-0.18 (-0.34 to -0.01)	0.034*
		R	-0.12 (-0.29 to 0.04)	0.129	-0.11 (-0.27 to 0.06)	0.198
**Lateral orbitofrontal**	Frontal	L	0.00 (-0.16 to 0.16)	0.993	0.00 (-0.16 to 0.17)	0.968
		R	-0.11 (-0.27 to 0.05)	0.189	-0.19 (-0.35 to -0.03)	0.020*
**Medial orbitofrontal**	Frontal	L	-0.16 (-0.32 to -0.00)	0.049*	-0.20 (-0.36 to -0.04)**	0.015*
		R	-0.16 (-0.32 to 0.01)	0.064	-0.29 (-0.45 to -0.12)**	0.001*
**Rostral middle frontal**	Frontal	L	-0.14 (-0.30 to 0.02)	0.095	-0.09 (-0.26 to 0.07)	0.263
		R	-0.12 (-0.28 to 0.04)	0.154	-0.19 (-0.35 to -0.03)	0.021*
**Superior parietal**	Parietal	L	-0.11 (-0.27 to 0.06)	0.196	-0.19 (-0.35 to -0.02)	0.026*
		R	0.03 (-0.14 to 0.20)	0.705	-0.09 (-0.26 to 0.09)	0.329
**Supramarginal**	Parietal	L	-0.00 (-0.17 to 0.16)	0.960	-0.03 (-0.19 to 0.14)	0.736
		R	-0.18 (-0.34 to -0.02)	0.028*	-0.15 (-0.31 to 0.01)	0.073

Footnote

Table showing the ROIs with associations for cognitive and language development with cortical surface area and cortical thickness, if either hemisphere had p<0.05. All linear regression models included child age and child sex as covariates; associations with surface area also included intracranial volume. The beta (standardised) regression coefficient represents the effect size or expected change in cognitive or language development (in standard deviations) with a one unit standard deviation change in the region-of-interest. ‡Right fusiform has n=143 for the cortical surface area calculation as one outlier was excluded. *p<0.05, **absolute beta coefficient>=0.20
